# Differentiating primary and secondary FSGS using non-invasive urine biomarkers

**DOI:** 10.1093/ckj/sfad296

**Published:** 2023-12-04

**Authors:** Lorenzo Catanese, Justyna Siwy, Ralph Wendt, Kerstin Amann, Joachim Beige, Bruce Hendry, Harald Mischak, William Mullen, Ian Paterson, Mario Schiffer, Michael Wolf, Harald Rupprecht

**Affiliations:** Department of Nephrology, Angiology and Rheumatology, Klinikum Bayreuth GmbH, Bayreuth, Germany; Kuratorium for Dialysis and Transplantation (KfH) Bayreuth, Bayreuth, Germany; Medizincampus Oberfranken, Friedrich-Alexander-University Erlangen-Nürnberg, Erlangen, Germany; Mosaiques Diagnostics GmbH, Hannover, Germany; Division of Nephrology, St. Georg Hospital Leipzig, Leipzig, Germany; Department of Nephropathology, Institute of Pathology, University Hospital Erlangen, Friedrich-Alexander University Erlangen-Nuremberg, Erlangen, Germany; Kuratorium for Dialysis and Transplantation (KfH) Renal Unit, Leipzig, Germany; Department of Internal Medicine II, Martin-Luther-University Halle/Wittenberg, Halle/Saale, Germany; Travere Therapeutics, San Diego, CA, USA; Mosaiques Diagnostics GmbH, Hannover, Germany; Institute of Cardiovascular and Medical Sciences, University of Glasgow, Glasgow, UK; Travere Therapeutics, San Diego, CA, USA; Department of Nephrology and Hypertension, University Hospital Erlangen, Friedrich-Alexander University (FAU) Erlangen-Nürnberg, Erlangen, Germany; Research Center on Rare Kidney Diseases (RECORD), University Hospital Erlangen, Erlangen, Germany; Travere Therapeutics, Dublin, Ireland; Department of Nephrology, Angiology and Rheumatology, Klinikum Bayreuth GmbH, Bayreuth, Germany; Kuratorium for Dialysis and Transplantation (KfH) Bayreuth, Bayreuth, Germany; Medizincampus Oberfranken, Friedrich-Alexander-University Erlangen-Nürnberg, Erlangen, Germany

**Keywords:** biomarkers, FSGS, non-invasive, peptides, proteomics, urine

## Abstract

**Background:**

Focal segmental glomerulosclerosis (FSGS) is divided into genetic, primary (p), uncertain cause, and secondary (s) forms. The subclasses differ in management and prognosis with differentiation often being challenging. We aimed to identify specific urine proteins/peptides discriminating between clinical and biopsy-proven pFSGS and sFSGS.

**Methods:**

Sixty-three urine samples were collected in two different centers (19 pFSGS and 44 sFSGS) prior to biopsy. Samples were analysed using capillary electrophoresis-coupled mass spectrometry. For biomarker definition, datasets of age-/sex-matched normal controls (NC, *n* = 98) and patients with other chronic kidney diseases (CKDs, *n* = 100) were extracted from the urinary proteome database. Independent specificity assessment was performed in additional data of NC (*n* = 110) and CKD (*n* = 170).

**Results:**

Proteomics data from patients with pFSGS were first compared to NC (*n* = 98). This resulted in 1179 biomarker (*P* < 0.05) candidates. Then, the pFSGS group was compared to sFSGS, and in a third step, pFSGS data were compared to data from different CKD etiologies (*n* = 100). Finally, 93 biomarkers were identified and combined in a classifier, pFSGS93. Total cross-validation of this classifier resulted in an area under the receiving operating curve of 0.95. The specificity investigated in an independent set of NC and CKD of other etiologies was 99.1% for NC and 94.7% for CKD, respectively. The defined biomarkers are largely fragments of different collagens (49%).

**Conclusion:**

A urine peptide-based classifier that selectively detects pFSGS could be developed. Specificity of 95%–99% could be assessed in independent samples. Sensitivity must be confirmed in independent cohorts before routine clinical application.

## Graphical Abstract

**Graphical Abstract:**
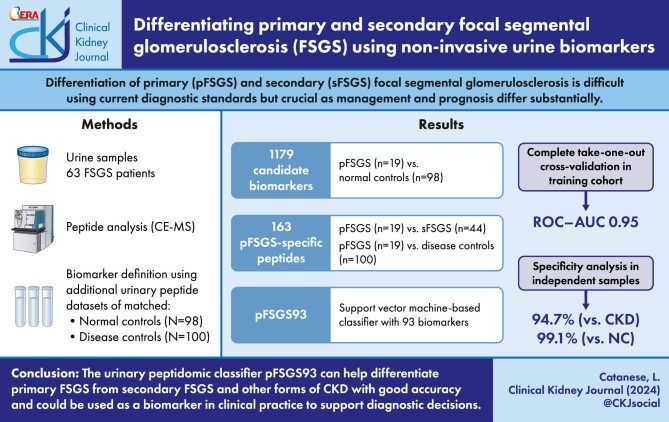

KEY LEARNING POINTS **What was known**:

- Defining the particular type of focal segmental glomerulosclerosis (FSGS) is important for choosing the optimal clinical management approach.
- Distinguishing between primary (p) FSGS and secondary (s) FSGS is essential to initiate necessary (and withhold unnecessary) immunosuppressive-based therapies.
- Urinary peptides have been shown to enable early detection of chronic kidney disease (CKD) with unsurpassed accuracy and also discrimination of FSGS from other CKD etiologies.

**This study adds**:

- Specific urinary peptides for pFSGS could be detected that showed significant and consistent dysregulation in pFSGS in comparison to sFSGS, normal controls and other CKD etiologies.
- The combination of the defined urinary peptide biomarkers into a classifier enables differentiation between pFSGS and sFSGS with good accuracy.

**Potential impact**:

- The presented non-invasive peptide-based differentiation could be used in clinical practice to support diagnostic decisions.
- The method would be of immediate value in instances where clinical presentation and histopathological findings are inconclusive to guide therapeutic decisions.
- Furthermore, the urinary biomarkers could support diagnostic and therapeutic decisions in cases with contraindications for kidney biopsy.

## INTRODUCTION

The lesion of focal segmental glomerulosclerosis (FSGS) represents a segmental increase in glomerular matrix with obliteration of the capillary lumina in at least one glomerulus of a renal biopsy. FSGS is a descriptive renal histologic lesion with diverse causes and pathogenicities that are linked by podocyte injury and depletion and lead to significant proteinuria. A total of 40% of nephrotic syndromes in adults and 20% of childhood nephrotic syndromes worldwide are caused by FSGS [1, 2].

The diagnosis of FSGS can be subdivided into genetic, primary (idiopathic, pFSGS), uncertain cause, and secondary (sFSGS) forms [3]. Defining the particular type of disease is important for choosing the right management approach. However, the diagnostic armamentarium available now lacks biomarkers of high accuracy.

Genetic forms may present as sporadic or familial disease with various inheritance patterns. Genetic FSGS is typically resistant to immunosuppressive therapy and does not recur in a renal transplant.

pFSGS is presumably caused by a circulating factor that causes injury to podocytes and thereby increases glomerular permeability [4], and it is characterized by heavy proteinuria. US data suggest that 40%–60% of FSGS patients progress to end-stage kidney disease [5]. Several molecules have been implicated in the pathogenesis of pFSGS, among them apolipoprotein A-1b (an isoform of Apo A1), cardiotrophin-like cytokine factor, anti-CD40 antibody, and soluble urokinase plasminogen activator receptor (suPAR) [6, 7]. The exact pathogenic mechanism, however, remains an unsettled issue. pFSGS may respond to corticosteroids, other immunosuppressive agents (i.e. calcineurin inhibitors), or plasmapheresis, although therapeutic response rates vary considerably with therapy-resistance rates being as high as 80% for steroids [8]. It has a high likelihood of recurrence after renal transplantation with reported recurrence rates of 30%–50% [9].

sFSGS includes maladaptive forms caused by a reduction in the number of functioning nephrons or a normal nephron mass subjected to abnormal stress (e.g. hypertension) resulting in an increase in single-nephron glomerular filtration rate, virus-associated forms (HIV, parvovirus B-19) [10–12], or drug-induced forms (pamidronate, interferons) [12–14], with the maladaptive form comprising the largest group. There are also susceptibility genes that confer an increased risk of FSGS. The best known of these are the G1 and G2 polymorphisms in the *APOL1* gene in patients with African ancestry, which are associated with an increased risk not only for FSGS but also for hypertensive nephropathy and HIV-associated nephropathy [15, 16]. There is potentially a genetic background that predisposes to the development of secondary, maladaptive FSGS, and the distinction between sFSGS and some genetic forms may not be so clear after all. For example, there are possibly causal genetic variants of collagen IV and Alport syndrome that have been associated with histopathological diagnoses of FSGS [17, 18].

As outlined above, therapeutic approaches to the various forms of FSGS vary considerably. Therefore, it is crucial to establish biomarkers and/or diagnostic algorithms that can reliably distinguish between the different forms, especially between pFSGS and sFSGS to avoid unnecessary, and not to withhold necessary, immunosuppressive-based therapies.

We have previously demonstrated that classifiers based on urinary peptides enable early detection in chronic kidney disease (CKD), guided by specific, molecular profiles [19, 20]. Further studies demonstrated the presence of specific biomarkers for FSGS that enable differentiation between FSGS and other CKD etiologies [21]. Within this project, capillary electrophoresis coupled mass spectrometry (CE-MS) was used for the definition of specific urinary proteins/peptides that discriminate primary from secondary FSGS.

The aim of our study was to establish a non-invasive urinary biomarker specific for pFSGS. For this purpose, we studied the urinary proteome and identified pFSGS-specific proteins/peptides and combined them into a classifier.

## MATERIALS AND METHODS

### Patient cohort

The urine samples were collected in two different centers in Germany from 2008 to 2021 and details are described in page 1 of Text S1 (see online supplementary material). Samples were collected on the day of the diagnostic kidney biopsy and before exposure to corticosteroids or other immunosuppressive therapies, with the following exceptions: one patient was permanently treated for rheumatoid arthritis with low dosage of corticosteroids, and in two patients the corticosteroid treatment was started before the sample collection (3 and 7 days, respectively).

The patient cohort of 63 FSGS patients was divided into primary (*n* = 19) and secondary (*n* = 44) FSGS forms. FSGS was only diagnosed if at least one FSGS lesion was present on light microscopy. All patients were recruited at time of diagnostic kidney biopsy. Immunofluorescence was performed on all patients evaluating IgA, IgG, IgM, C1q, and C3c in the mesangial and GBM compartment of the glomeruli. Electron microscopy (EM) to determine the pattern and amount of foot process effacement (FPE) was performed in all patients. The primary distinction between pFSGS and sFSGS was based on EM findings (>80% FPE) and clinical characteristics, mainly the presence of nephrotic syndrome (nephrotic proteinuria of >3.5 g/d, hypoalbuminemia of <3500 mg/dl, and edema) as well as absence of secondary causes that were ruled out by utilizing clinical diagnostic procedures [22]. Genetic analysis was not performed. Serum levels of creatinine for eGFR calculation, albumin, cholesterol, proteinuria, and all other laboratory parameters were obtained at time of biopsy. The characteristics of the patients per group are given in Table 1.

**Table 1:** Characteristics of patients at diagnosis used for biomarker definition.

	Primary FSGS	Secondary FSGS		Normal control		CKD	
	*n* = 19	*n* = 44	*P*-value	*n* = 98	*P*-value	*n* = 100	*P*-value
Sex, male, *n* (%)	13 (68.4)	30 (68.2)	0.7824	73 (74.5)	0.7913	73 (73.0)	0.8972
Age, years	46.3 (16.8)	57.6 (16.7)	0.016	44.7 (15.4)	0.6878	45.5 (14.7)	0.8311
BMI, kg/m^2^	31.0 [27.0–33.3]	28.7 [24.5–31.3]	0.1493	NA	NA	NA	NA
BP syst., mmHg	140 [131–147]	140 [124–150]	0.7698	NA	NA	NA	NA
BP diast., mmHg	85 [73–90]	80 [70–90]	0.6499	NA	NA	NA	NA
eGFR (CKD-EPI), ml/min/1.73 m²	56.0 [37.4–94.9]	31.1 [18.0–43.9]	0.0008	88.4 [72.6–114.8]	0.0054	40.7 [20.9–76.8]	0.0414
Proteinuria, g/d	8.03 [5.60–11.11]	2.56 [1.3–4.00]	<0.0001	0.01 [0.01–0.19]	<0.0001	2.00 [0.69–5.80]	<0.0001
IFTA, %	12.4 (11.9)	28.3 (18.9)	0.0013	NA	NA	16.6 (18.9)	0.3757
Diabetes, *n* (%)	4 (21)	9 (20)	0.7754	24 (24)	0.978	15 (15)	0.750
Serum cholesterol, mg/dl	297 [220–381]	200 [158–243]	0.0004	NA	NA	NA	NA
Serum albumin, g/dl	2.88 [1.84–3.41]	4.24 [3.62–4.56]	<0.0001	NA	NA	NA	NA
Nephrotic-range proteinuria, *n* (%)	14 (73.7)	14 (31.8)	0.0052	0 (0)	<0.0001	32 (32)	0.0016
Nephrotic syndrome, *n* (%)	13 (68.4)	4 (9.1)	<0.0001				
ACEi/ARB treatment, *n* (%)	16 (84.2)	31 (70.5)	0.4032	NA	NA	NA	NA

Data are presented as mean (standard deviation), median [interquartile range], or *n* (%). *P*-values are given for pFSGS versus the respective group and are calculated using Student's *t* test, Mann‐Whitney *U* test, and the χ^2^ test for continuous, non-normal continuous, and categorical variables, respectively. ACEi: angiotensin-converting-enzyme inhibitor; ARB: angiotensin receptor blocker; BMI: body mass index; BP: blood pressure; eGFR: estimated glomerular filtration rate; IFTA: interstitial fibrosis and tubular atrophy; NA: not available.

### Sample preparation and CE-MS analysis

The samples were transferred on dry ice and thawed immediately before use and prepared as described before [23]. Briefly, 0.7 mL of urine were diluted with 0.7 mL of a solution containing 2 M urea (VWR Chemicals, Leuven, Belgium), 10-mM NH4OH (Merc KGaA, Darmstadt, Germany), and 0.02% SDS (Carl Roth GmbH, Karlsruhe, Germany). The samples were ultrafiltered using a Centrisart ultracentrifugation filter device (20 kDa molecular weight cutoff; Sartorius, Goettingen, Germany). Subsequently, 1.1-mL filtrate was obtained and applied onto a PD-10 desalting column (GE Healthcare Bio Sciences, Uppsala, Sweden) equilibrated in 0.01% aqueous NH_4_OH. Finally, the eluate was lyophilized and stored at 4°C prior to resuspension in high-performance liquid chromatography (HPLC)-grade water for CE-MS.

The prepared samples were analysed using a P/ACE MDQ CE coupled on-line to a MicrOTOF II MS, following the sample protocol as previously described [24]. Peptide detection and quantification are described in detail in pages 1 and 2 of Text S1 (see online supplementary material). All detected peptides are deposited, matched, and annotated in a Microsoft SQL database allowing further statistical analysis.

### Sequencing of peptides

The amino acid sequence information was obtained using CE-MS/MS or liquid chromatography (LC)-MS/MS, as published [25] and summarized in page 2 of Text S1 (see online supplementary material).

### Statistical analysis

The demographic and clinical parameters are presented in Table 1 as mean ± standard deviation (SD) or median [interquartile range (IQR)] if non-normally distributed and n (%) if categorical. *P*-values were calculated using MedCalc software (version 12.1.0.0; MedCalc Software, Mariakerke, Belgium) using Student's *t* test, Mann‐Whitney *U* test and χ^2^ test for continuous, non-normal continuous, and categorical variables, respectively.

The biomarkers were defined using statistical analysis performed using R-based statistic software and combined using support vector machine (SVM) algorithm. Details are described in pages 2 and 3 of Text S1 (see online supplementary material).

### Extraction of additional CE-MS datasets

Additional CE-MS datasets were used as controls to increase the specificity of the generated classifier. These were extracted from the urinary proteome database [26] that currently includes >85 000 datasets processed and normalized as described above. This approach results in highly comparable datasets with no detectable batch effects [27, 28].

For the biomarker definition, datasets of 98 normal control (NC) subjects and from 100 patients with different CKD etiologies (Table 2) were extracted and matched to the pFSGS patients for age and sex. Characteristics of these patients are shown in Table 1.

**Table 2:** List of selected age- and sex-matched patients with different CKD etiologies other than FSGS.

	Other CKD	N
		
AMYLOID	Amyloidosis	4
ATN	Acute tubular necrosis	8
C3MPPI_GP	Membranoproliferative GN. C3-GN. Postinfectious GN	3
CAST	Myeloma cast nephropathy	2
COLIVAD	Collagen IV associated diseases	6
DNP	Diabetic nephropathy with nodular nephrosclerosis	10
HINP	Hypertensive ischemic nephropathy	13
IGANP	IgA nephropathy	16
IGAPSH	Henoch-Schoenlein purpura (IgA vasculitis)	5
INTN	Interstitial nephritis	6
LN	Lupus nephritis	4
MCGN	Minimal change glomerulopathy	5
MEMGN	Membranous nephropathy	9
VASCular	Thrombotic microangiopathy (cholesterol embolism)	2
VASCulitis	Vasculitis	7

For independent specificity analysis, additional CE-MS data of independent NC subjects (*n* = 110) and patients with different CKD etiologies (*n* = 170) were used (Table 3).

**Table 3:** List of additional independent datasets of patients with different CKD etiologies other than FSGS.

	Other CKD	N
		
AMYLOID	Amyloidosis	1
ATN	Acute tubular necrosis	1
DNP	Diabetic nephropathy with nodular nephrosclerosis	66
HINP	Hypertensive ischemic nephropathy	9
IGANP	IgA nephropathy	63
INTN	Interstitial nephritis	1
LN	Lupus nephritis	3
MCGN	Minimal change glomerulopathy	6
MEMGN	Membranous nephropathy	6
MPGN	Membranoproliferative glomerulonephritis	5
VASCulitis	Vasculitis	9

## RESULTS

Clinical and histopathological findings led to the classification of the FSGS patients into pFSGS and sFSGS. pFSGS was only diagnosed when FPE was >80% in EM. Immunochemistry was negative or unspecific in all patients except for three patients with IgA nephropathy. Two of these patients were classified as sFSGS, while one patient was still classified as pFSGS because of severe FPE and nephrotic syndrome.

Urinary proteome analysis was performed for patients with pFSGS (*n* = 19) and sFSGS (*n* = 44). Details of FSGS patients and the CKD and NC groups are shown in Table 1 and are described further in pages 3 and 4 of Text S1 (see online supplementary material).

**Figure 1:**
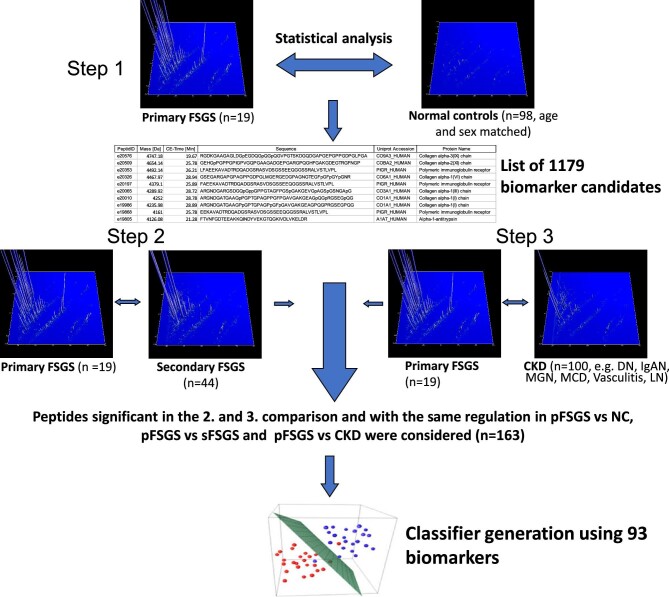
Study workflow. pFSGS-specific biomarkers were defined in three steps. In the first step, the urinary peptide data of pFSGS were compared to normal controls. For further analysis only peptides with a *P*-value <0.05 (adjusted for multiple testing) were considered (*n* = 1179). These potential biomarkers were investigated for significant differences and identical directional change (up- or downregulated) in two additional comparisons: pFSGS versus sFSGS, and pFSGS versus other CKD etiologies. This resulted in a final list of 163 pFSGS-specific peptide biomarkers that were combined into a high-dimensional classifier using support vector machine. For training of the classifier pFSGS versus sFSGS data were used. The classifier was optimized using a take-one-out procedure, which resulted in exclusion of 70 peptides. The final classifier, pFSGS93, consisted of 93 peptides.

The biomarker definition was performed in three steps (Fig. 1). Because the biggest difference in the urinary peptide content was expected between the pFSGS and NC groups, this comparison was performed in a first step. For the statistical analysis, the Wilcoxon rank-sum test was applied, and the *P*-values were adjusted for multiple testing. For further analysis, only peptides were considered with an adjusted *P*-value <0.05. This resulted in a list of 1179 potential biomarker candidates, differentiating pFSGS from NC.

In the next step, these 1179 potential biomarkers were investigated for significant differences and identical directional change (up- or downregulated) in two additional comparisons: pFSGS (*n* = 19) versus sFSGS (*n* = 44), and pFSGS (*n* = 19) versus CKD of other etiologies (*n* = 100). This resulted in a final list of 163 pFSGS-specific peptide biomarkers that passed all statistical tests and showed a change in comparison to the other groups.

**Figure 2:**
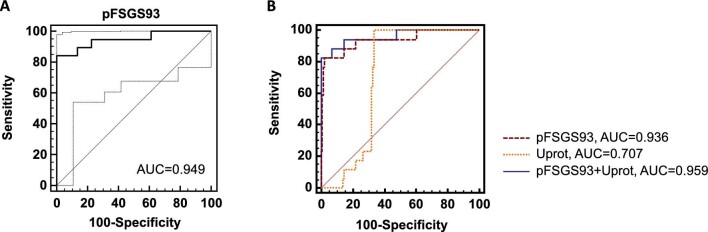
ROC curves for the classification of the complete take-one-out cross-validated training cohort. (**A**) ROC curve of primary (*n* = 19) versus secondary (*n* = 44) FSGS patients. (**B**) Comparison of ROC curves for the discrimination of pFSGS versus sFSGS for which the proteinuria and eGFR values were available for pFSGS93 classifier, eGFR, proteinuria and the combination of all three parameters.

Datasets of patients with pFSGS (*n* = 19) and sFSGS (*n* = 44) were used for the model development. The selected biomarker candidates were combined into an SVM-based classifier. The classifier was optimized using a take-one-out procedure. Excluded were 70 peptides. Further reduction of the number of peptides also resulted in reduced performance in the complete take-one-out cross-validation. The final classifier based on 93 biomarkers, called pFSGS93, showed an area under the receiver operating characteristic (ROC) curve (AUC) of 0.95 (95%CI 0.88–1.00) when testing applying complete take-one-out cross-validation in the training cohort (as no additional samples of FSGS patients for testing were available; Fig. 2A). The definition of the best suitable diagnosis threshold using Youden index resulted in a cutoff of −0.001 with sensitivity of 84.2% and specificity of 100%. The urinary peptide biomarkers included in the model are listed in Table 4.

**Table 4:** List: of 93 pFSGS-specific peptides.

Peptide ID	Amino acid sequence	Protein name	Step 1: pFSGS vs NC adj. *P*-value	Step 2: pFSGS vs sFSGS Wilcoxon *P*-value	Step 3: pFSGS vs CKD Wilcoxon *P*-value	Mean int. pFSGS	Mean int. NC	Mean int. sFSGS	Mean int. CKD
e09695	LLSPYSYSTTAVVTNPKE	Transthyretin	5.31E-18	3.25E-03	3.61E-05	61 777.16	0.2	8099.46	1777.77
e02155	EAQLPVIENK	Plasminogen	5.31E-18	3.51E-04	1.52E-06	2049.89	0	342.39	382.34
e09024	NTKSPLFMGKVVNPTQK	Alpha-1-antitrypsin	5.31E-18	2.06E-03	1.10E-04	103 683.25	0	1494.92	3698.6
e00846	GKVVNPTQK	Putative alpha-1-antitrypsin-related protein	5.31E-18	2.76E-02	7.23E-03	2922.6	0	1524.77	822.9
e12741	MIEQNTKSPLFMGKVVNPTQK	Alpha-1-antitrypsin	7.78E-17	2.23E-03	2.60E-05	1 382 750	624.02	560 954.71	114 818.64
e20576	RGDKGAAGAGLDGpEGDQGpQGpQGVPGTSKDGQDGAPGEPGPPGDPGLPGA	Collagen alpha-3(IX) chain	7.78E-17	5.89E-05	1.18E-08	2340.53	0	376	225.03
e10918	EQNTKSPLFMGKVVNPTQK	Alpha-1-antitrypsin	7.78E-17	9.51E-03	1.40E-03	33 165.64	0	2883.03	1968.78
e13568	GpTGYKGEQGEVGKDGEKGDPGPpGP	Collagen alpha-3(IX) chain	8.94E-16	1.49E-02	3.14E-03	5539.71	1.66	3930.26	2352.2
e10047	QNTKSPLFMGKVVNPTQK	Alpha-1-antitrypsin	9.82E-16	2.12E-05	5.40E-05	2640.35	0	122.88	336.92
e08905	PMSIPPEVKFNKPFVF	Alpha-1-antitrypsin	9.82E-16	1.54E-02	7.22E-03	14 615.91	0	9902.5	4673.23
e11723	IEQNTKSPLFMGKVVNPTQK	Alpha-1-antitrypsin	2.86E-15	8.41E-03	1.91E-04	294 372.04	67.93	89 420.89	36 286.93
e11839	IEQNTKSPLFmGKVVNPTQK	Alpha-1-antitrypsin	4.90E-15	2.15E-02	1.69E-03	18 139.5	23.29	2230.73	5906.82
e10328	LEAIPMSIPPEVKFNKPF	Alpha-1-antitrypsin	1.51E-14	2.58E-02	1.01E-03	24 702.08	0	9799.42	3928.78
e12127	LREGETKAVKTVRTPGAAANLE	Alpha-1B-glycoprotein	8.75E-14	2.85E-02	2.53E-03	77 915.23	107.9	9514.84	7899.69
e03374	HSVSPPPPYPGH	Transmembrane gamma-carboxyglutamic acid protein 4	2.19E-13	2.69E-02	8.70E-04	16 653.86	0.24	5372.26	829.79
e15341	LRTLNQPDSQLQLTTGNGLFLSEGLK	Alpha-1-antitrypsin	2.19E-13	8.98E-03	3.59E-05	9599.12	0	1369.04	742.25
e15184	EDPQGDAAQKTDTSHHDQDHPTFNK	Alpha-1-antitrypsin	2.19E-13	9.86E-03	3.18E-03	48 908.65	0	598.53	455.38
e04671	LSALEEYTKKLN	Apolipoprotein A-I	2.19E-13	2.99E-02	9.58E-06	4955.61	0	1511.53	483.79
e13894	SQLSNNAKEAVEHLQKSELTQQL	Apolipoprotein A-IV	2.19E-13	4.03E-02	9.28E-03	5691.78	0	1576.26	1139.96
e03221	SIPPEVKFNKP	Alpha-1-antitrypsin	2.19E-13	4.75E-02	8.04E-04	138 403.47	0	5378.5	4523.59
e03394	LMIEQNTKSPL	Alpha-1-antitrypsin	2.22E-13	4.48E-02	6.75E-04	15 243.59	20.29	11 393.82	2103.25
e10252	PGNEGLDGpRGDpGQpGPPGE	Collagen alpha-3(IV) chain	6.88E-13	8.41E-06	3.40E-05	2467.77	13.48	364.71	733.59
e12049	LEAIPMSIPPEVKFNKPFVF	Alpha-1-antitrypsin	2.97E-12	1.10E-02	2.50E-03	121 762.46	46.18	112 295.62	64 233.39
e15418	LENEDRRSASLHLPKLSITGTYDLK	Alpha-1-antitrypsin	2.97E-12	2.87E-02	1.20E-02	32 087.05	0	763.9	977.5
e01177	LTEKRMDK	Complement C3	3.59E-11	3.32E-04	5.78E-04	258.16	0	12.12	37.5
e10994	LEAIPMSIPPEVKFNKPFV	Alpha-1-antitrypsin	3.59E-11	1.95E-03	1.01E-03	8309.61	0	476.55	4581.16
e19805	FTVNFGDTEEAKKQINDYVEKGTQGK IVDLVKELDR	Alpha-1-antitrypsin	3.59E-11	1.70E-02	7.63E-03	6062.66	0	274.88	222.01
e19801	GEPGRDGVPGGPGMRGMPGSPGGP GSDGKPGPpGSQGESGRpGpP	Collagen alpha-1(III) chain	3.71E-11	1.70E-02	7.31E-03	51 235.79	31.42	4342.35	6599.49
e07561	LSPYSYSTTAVVTNPK	Transthyretin	4.58E-11	4.21E-02	1.43E-02	1135.71	3.73	174.88	73.4
e13512	EMLIAGFLYVADLENMVQYRR	E3 ubiquitin-protein ligase RNF146	4.96E-10	6.04E-05	6.62E-06	464.76	0	9.88	35.36
e13364	DNWDSVTSTFSKLREQLGPVTQ	Apolipoprotein A-I	4.96E-10	2.37E-02	4.78E-04	3488.21	0	145.65	169.55
e03750	SQRFPKAEFAE	Albumin	5.09E-09	7.09E-03	1.35E-03	1562.72	1.96	100.32	100.25
e19009	GPAGpPGFPGAVGAKGEAGPQGPR GSEGPQGVRGEPGPPGPA	Collagen alpha-1(I) chain	6.65E-09	1.11E-03	1.07E-06	259.1	0	216.05	14.68
e11174	KGNSGEPGApGSKGDTGAKGEpGP	Collagen alpha-1(I) chain	6.65E-09	3.16E-02	7.90E-03	5812.98	0	29.98	29.03
e13118	PpGADGQPGAKGEpGDAGAKGDAGP PGP	Collagen alpha-1(I) chain	6.65E-09	4.27E-02	2.00E-03	178.1	0	33.7	12.14
e08418	ApGAPGGKGDAGApGERGpPG	Collagen alpha-1(III) chain	2.70E-08	3.45E-02	1.53E-02	1597.22	9.65	473.25	427.55
e18377	FAEEKAVADTRDQADGSRASVDSGSS EEQGGSSRA	Polymeric immunoglobulin receptor	2.93E-08	1.27E-03	7.17E-03	120.54	2129.92	487.65	672.89
e00067	HAGAQGppG	Collagen alpha-1(III) chain	5.27E-08	2.50E-02	1.38E-02	2185.26	5.25	343.93	232.63
e08498	TGPIGPpGPAGAPGDKGESGP	Collagen alpha-1(I) chain	7.00E-08	3.59E-02	6.94E-03	483.42	17.05	214.95	128.09
e07720	LGApGPSGARGERGFpGE	Collagen alpha-1(I) chain	8.49E-08	4.40E-03	2.21E-04	7208.41	15.83	2863.11	435.66
e11311	FLEAIPMSIPPEVKFNKPF	Alpha-1-antitrypsin	8.49E-08	7.86E-03	1.30E-03	1986.44	0	77.65	108.88
e01266	KRPPGFSPF	Kininogen-1	8.49E-08	4.16E-02	3.88E-02	580.54	0	42.4	37.62
e16540	GPAGAAGARGNDGQPGPAGPPGP VGpAGGpGFpGAPG	Collagen alpha-1(II) chain	1.63E-07	1.33E-02	3.60E-02	45.75	588.36	133.05	164.41
e01814	VDVLKDSGRD	Apolipoprotein A-I	2.47E-07	2.76E-02	1.80E-02	404.76	1.59	67.03	76.27
e18092	NTGApGSpGVSGpKGDAGQpGEKGS pGAQGpPGAPGpLG	Collagen alpha-1(III) chain	2.91E-07	8.92E-03	5.07E-05	1632.49	4463.37	3387.39	3789.74
e09154	NTKSPLFmGKVVNPTQK	Alpha-1-antitrypsin	7.10E-07	2.69E-02	4.32E-02	3958.67	2.85	148.08	700.64
e09634	FTDTEDPAKFKMKYWG	Retinol-binding protein 4	1.05E-06	3.84E-02	7.40E-05	1324.44	5.57	347.35	97.59
e00992	VRGEpGPpGP	Collagen alpha-1(I) chain	1.15E-06	4.86E-03	6.79E-03	231.09	0.27	179.49	47.78
e12878	WYKGEKKLFNGQQGIIIQNF	Neuronal growth regulator 1	1.28E-06	7.31E-03	1.95E-04	1017.75	0.73	11.22	64.95
e01727	DLRDKVNSF	Apolipoprotein A-IV	3.31E-06	1.02E-02	1.32E-02	2390.71	1.38	74.39	132.17
e00764	GFQIVHSLG	Tubulin beta-1 chain	3.78E-06	2.47E-02	9.21E-04	566.57	1.69	70.3	24.77
e00428	VENDEMPA	Albumin	4.06E-06	8.54E-04	9.30E-03	40.23	0.47	2.87	11.4
e17280	AAGEpGKAGERGVpGPpGAVGPA GKDGEAGAQGPPGP	Collagen alpha-1(I) chain	6.81E-06	2.33E-02	6.28E-03	427.21	1371.46	764.55	882.75
e20010	ARGNDGATGAAGpPGPTGPAGPP GFPGAVGAKGEAGpQGpRGSEGpQG	Collagen alpha-1(I) chain	6.94E-06	1.11E-02	1.43E-02	163.37	982.53	359.05	376.93
e20065	ARGNDGARGSDGQpGppGPPGTAG FPGSpGAKGEVGpAGSpGSNGApG	Collagen alpha-1(III) chain	7.11E-06	5.26E-04	6.34E-04	1103.05	4464.53	3764.46	3255.42
e17288	GPpGESGREGAPGAEGSpGRDGS pGAKGDRGETGp	Collagen alpha-1(I) chain	1.61E-05	4.40E-03	8.81E-03	821.76	3027.12	2692.51	2135.13
e11006	KGpSGVpGSAGPEGEPGLIGPpGP	Collagen alpha-5(IV) chain	3.10E-05	5.67E-04	4.62E-02	290.07	35.59	99.67	118.57
e01243	IEQNTKSPL	Alpha-1-antitrypsin	3.39E-05	1.65E-02	4.22E-03	5524.23	26.1	410.64	884.34
e00899	IDGRPGPIGP	Collagen alpha-2(I) chain	4.67E-05	1.29E-02	3.60E-03	232.95	2.02	179.16	75.9
e11087	KGDpGDVGGPGpPGASGEpGAPGPP	Collagen alpha-3(V) chain	5.13E-05	7.31E-03	1.54E-02	50.96	8.36	4	20.86
e20197	FAEEKAVADTRDQADGSRASVDSG SSEEQGGSSRALVSTLVPL	Polymeric immunoglobulin receptor	5.64E-05	6.16E-03	7.17E-03	71.09	1317.95	698.13	715.86
e00028	GEPGPEGPA	Collagen alpha-2(V) chain	1.41E-04	1.05E-02	5.43E-03	24.88	0.13	6.58	3.49
e00259	ApGERGPpG	Collagen alpha-2(IV) chain	1.67E-04	2.06E-02	4.33E-03	660.48	197.17	448.43	311.1
e17207	DAAQKTDTSHHDQDHPTFNKITPN L AEFA	Alpha-1-antitrypsin	2.58E-04	2.34E-03	1.67E-02	8.18	287.63	171.56	188.34
e15333	GAPGpQGFQGppGEPGEPGASGP MGPRGPPG	Collagen alpha-1(I) chain	3.39E-04	2.70E-02	5.99E-04	388.7	1002.11	780.91	1000.98
e07640	GPpGpPGKNGDDGEAGKPG	Collagen alpha-1(I) chain	3.67E-04	4.39E-02	1.74E-02	713.19	47.85	178.42	232.26
e08582	VGEPGpAGSKGESGNKGEpG	Collagen alpha-2(I) chain	6.28E-04	3.80E-02	1.39E-03	1226.89	18.61	507.44	94.6
e19868	EEKAVADTRDQADGSRASVDSGSS EEQGGSSRALVSTLVPL	Polymeric immunoglobulin receptor	1.00E-03	4.04E-02	6.62E-03	978.17	2223.26	1359.54	1335.23
e05114	SpGSpGPDGKTGPPGp	Collagen alpha-1(I) chain	1.10E-03	4.02E-02	4.94E-03	32 505.02	24 548.73	27 897.72	26 264.19
e20326	GSEGARGAPGPAGPPGDPGLMGER GEDGPAGNGTEGFpGFpGYpGNR	Collagen alpha-1(VI) chain	1.55E-03	1.48E-02	2.16E-02	49.64	299.97	157.51	151.97
e18770	VAMPGGPGTPGFpGERGNSGEHGE IGLpGLpGLPGTPGN	Collagen alpha-3(IV) chain	1.77E-03	4.56E-02	2.81E-02	17.47	100.91	72.91	75.12
e10976	PpGADGQpGAKGEQGEAGQKGDA	Collagen alpha-1(II) chain	1.97E-03	4.84E-02	9.37E-03	3.79	91.09	268.3	60.95
e05274	DDGEAGKpGRpGER	Collagen alpha-1(I) chain	2.23E-03	3.35E-02	1.34E-02	286.81	837.2	609.77	667.83
e12235	GQNGEpGGKGERGApGEKGEGGPpG	Collagen alpha-1(III) chain	2.25E-03	2.31E-03	1.85E-02	398.35	418.95	1635.56	566.48
e15580	GpSGpVGpPGLAGERGEQGPpG PTGFQGLPG	Collagen alpha-2(V) chain	2.40E-03	1.14E-02	1.17E-03	56.81	204.96	250.46	345.18
e11008	pPGEEGKRGPRGDpGTVGPpGP	Collagen alpha-2(V) chain	3.95E-03	3.73E-03	2.73E-03	762.72	159.45	517.11	415.02
e20509	GEHGpPGPPGPIGPVGQPGAAGAD GEPGARGPQGHFGAKGDEGTRGFN GP	Collagen alpha-2(XI) chain	5.45E-03	3.91E-03	9.83E-03	1393.6	2247.7	3679.08	3611.95
e10668	GpPGEAGKpGEQGVpGDLGAPGp	Collagen alpha-1(I) chain	6.06E-03	1.20E-02	6.39E-04	1184.73	1748.17	2088.63	2599.87
e19986	ARGNDGATGAAGPpGPTGPAGPpGF pGAVGAKGEAGPQGPRGSEGPQG	Collagen alpha-1(I) chain	8.18E-03	7.73E-04	4.23E-03	55.4	351.04	296.37	332.66
e14204	QGPPGPSGEEGKRGpNGEAGSAGp pGPPG	Collagen alpha-2(I) chain	1.01E-02	9.47E-03	9.00E-03	322.45	539.18	664.62	595.93
e03194	DGVPGKDGPRGPT	Collagen alpha-1(III) chain	1.37E-02	4.81E-03	4.85E-02	4119.62	201.69	996.61	404.43
e17923	EVGPAGSPGSNGAPGQRGEpGPQG HAGAQGPPGPPGIN	Collagen alpha-1(III) chain	1.52E-02	1.85E-02	8.80E-03	33.06	18.46	20.62	24.9
e02474	FLGKVVNPTEA	Thyroxine-binding globulin	1.77E-02	1.58E-02	5.90E-04	841.16	25.09	11.97	19.64
e10838	GpTGpIGPpGpAGQPGDKGEGGAP	Collagen alpha-1(III) chain	2.13E-02	1.54E-03	3.05E-03	453.32	53.37	250.64	76.03
e01100	DRGEpGPpGP	Collagen alpha-1(VII) chain	2.17E-02	3.92E-02	2.26E-03	570.31	111.37	424.11	102.92
e02794	DGPAGApGTpGPQG	Collagen alpha-1(I) chain	2.23E-02	1.92E-04	2.15E-02	146.06	56.12	15.77	65.58
e04013	GLDGpRGDPGQPGP	Collagen alpha-3(IV) chain	2.67E-02	5.05E-03	1.88E-03	411.87	70.36	100.15	87.37
e01147	SVIDQSRVL	Uromodulin	2.70E-02	2.46E-02	5.22E-03	179.72	38.37	34.89	43.84
e10452	NRNPGSSGTGGTATWKPGSSGP	Fibrinogen alpha chain	2.71E-02	1.84E-02	2.38E-02	259.02	323.99	1091.77	602.73
e19324	EPGSAGPQGPPGPSGEEGKRGPNGEA GSAGPPGppGLRGSpGS	Collagen alpha-2(I) chain	3.46E-02	4.00E-02	1.97E-02	314.8	582.78	1596.32	1995.09
e00837	KGDTGPpGPQ	Collagen alpha-1(III) chain	3.51E-02	1.56E-03	8.42E-06	51.47	14.34	14.83	7.15
e01377	SGSVIDQSRV	Uromodulin	3.58E-02	1.63E-02	6.25E-04	8474.79	684.39	765.24	887.03
e20353	LFAEEKAVADTRDQADGSRASVDSG SSEEQGGSSRALVSTLVPL	Polymeric immunoglobulin receptor	3.63E-02	8.98E-03	1.10E-02	92.35	235.84	1437.1	782.77

Given are the peptide IDs, amino acid sequences (with p: hydroxyproline, m: oxidized methionine), and the parental protein names. In addition, given are the *P*-values for the individual statistical steps and the mean signal intensities (mean int.) for the patients’ groups. Peptides are ordered by *P*-values obtained in the first statistical analysis (step 1).

### Urinary peptide biomarkers

Most prominently, fragments of different collagen proteins (*n* = 46, 49.5%) showed different abundance when comparing pFSGS with sFSGS, NC and other etiologies and formed the majority of pFSGS93. Furthermore, decreased abundance of peptides from polymeric immunoglobulin receptor (PIGR) and increased abundance of alpha-1-antitrypsin, transthyretin, and uromodulin peptides in pFSGS were observed. In addition, complement C3 peptide was more abundant in the pFSGS group in comparison to sFSGS, NCs, and other CKD etiologies. Peptides with the most significant differences in the comparison pFSGS versus NCs were fragments of blood proteins like transthyretin, alpha-1-antitrypsin, and fibrinogen that were increased in the pFSGS patients. The most significant peptides in the comparison of pFSGS versus sFSGS were upregulated fragments of different collagen fragments, alpha-1-antitrypsin, E3 ubiquitin-protein ligase RNF146, complement C3, and plasminogen and downregulated a fragment of PIGR.

### Analysis of covariables and nomogram generation

Multiple regression was used to estimate whether additional parameters are associated with the diagnosis of pFSGS. The data of pFSGS, sFSGS, and additional 100 CKD patients were used. The following parameters were analysed: pFSGS93, sex, age, proteinuria, eGFR, and interstitial fibrosis and tubular atrophy (IFTA). Only pFSGS93 and proteinuria remained significant (*P* = 0.007 and *P* = 0.0143, see also page 4 of Text S1 (see online supplementary material)). The comparison of the ROC analysis is shown in Fig. 2B. The pFSGS93 resulted in a significantly higher AUC of 0.94 (95%CI 0.86–1.00) than proteinuria (AUC = 0.71; 95%CI 0.63–1.00). These two parameters were combined in a nomogram. The combination of pFSGS93 and proteinuria resulted in a significantly higher AUC = 0.96 (95%CI 0.90–1.00) in comparison to proteinuria alone. However, in comparison to pFSGS93 alone, no significant improvement could be reached.

### Specificity analysis

Specificity of the pFSGS93 was investigated in an additional independent set of NC (*n* = 110) and CKD with other etiologies (*n* = 170). Using the specific cutoff of −0.001, 161 of the 170 (94.7%) patients with other CKD etiologies, and 109 of the 110 NC were (99.1%) correctly classified as no pFSGS.

## DISCUSSION

A strict distinction between pFSGS and sFSGS is not possible, but several approaches have been made to discern these two entities [29].

### Range of proteinuria and clinical history

pFSGS usually presents with nephrotic-range proteinuria (>3.5 g/d) with nephrotic syndrome, hypertension, microhematuria [30] and a rapid onset of disease. sFSGS patients can present with a broad range of proteinuria (including nephrotic range) but, in general, do not develop nephrotic syndrome. Proteinuria frequently shows a slow increase over time [31]. Risk factors like obesity, vesicoureteral reflux, renal agenesis, reduced nephron mass, or infection may be present.

### Histological findings

It is essential to obtain a representative biopsy specimen with at least 10 glomeruli, both cortical and juxtamedullary, as sclerotic lesions occur earlier in the latter.

Effacement of the epithelial foot processes of glomerular podocytes is thought to be diffuse and extensive in pFSGS [32]. In patients with pFSGS, 64.9% of podocytes showed diffusely fused foot processes, and 35.1% showed focal fusion. In sFSGS, the percentage of FPE typically ranges from 25% to 40% [33–36], whereas it ranged from 65% to 100% in series of pFSGS [33, 35, 36].

According to their findings, Sethi *et al.* [37]. suggest that dividing FSGS into presence or absence of nephrotic syndrome together with the degree of FPE on electron microscopy can therefore be used to facilitate the distinction between pFSGS and sFSGS.

There are, however, forms of FSGS with widespread FPE, like pamidronate-induced sFSGS [13] and also genetic FSGS [38, 39]. These findings show that FPE is important in distinguishing pFSGS from other forms of FSGS but cannot serve as a single biomarker for pFSGS. Therefore, the need for better biomarkers is crucial.

### Biomarkers

Advances have been made in the discovery of biomarkers for glomerular diseases, including the discovery of the M-type phospholipase A2 receptor, thrombospondin type-1 domain-containing 7A, neural epidermal growth factor-like 1 protein and others as target antigens in many patients with membranous GN [40, 41] or galactose-deficient IgA1 and antiglycan response in IgA nephropathy [42]. Further studies in FSGS are described in pages 5–7 of Text S1 (see online supplementary material). In summary, no diagnostic biomarker has been established to distinguish pFSGS from sFSGS.

PIGR peptide fragments showed decreased abundance in pFSGS. Different urinary PIGR signals have recently been shown to be associated with different CKD etiologies. Furthermore, PIGR seemed to be inversely correlated with eGFR in a large cohort [43]. Other authors have also shown different PIGR expression in kidney tissues in different disease etiologies [44]. The identification of clear pathomechanisms or functional links to disease formation remain unclear, though. Differences in urinary abundance in alpha-1-antitrypsin and uromodulin have previously been suggested for distinction between minimal change glomerulopathy (MCGN) and FSGS disregarding the different subcategories of FSGS [45]. The pathogenetic role of alpha-1-antitrypsin in glomerular diseases with nephrotic proteinuria remains unclear. Candiano *et al.* found different fragments of albumin and alpha-1-antitrypsin to be associated with different entities of nephrotic syndromes. The authors suggested that disease-specific protease cleaving might occur in the urine and that might be helpful for disease classification [46]. Uromodulin or Tamm-Horsfall protein is a kidney-specific protein synthesized on the epithelial cells of the thick ascending limb (TAL) of Henle's loop and the most abundant urinary protein in healthy individuals [47]. Interestingly, Chun *et al.* found uromodulin mutations in a rather large subgroup of sFSGS patients that were then classified as Autosomal Dominant Tubulointerstitial Kidney Disease (ADPKD), which has been previously linked to uromodulin gene variants [48]. These findings are broadly consistent with our findings of different urinary uromodulin abundance in pFSGS, sFSGS, and healthy controls. The finding of complement C3 being differently abundant might indicate the involvement of complement pathways in FSGS, which has previously been suggested by Thurman *et al.* [49].

The results reported here indicate that CE-MS technology can be applied for the identification of urinary peptides significantly associated with pFSGS. Moreover, these biomarkers combined in a classifier enable discrimination of pFSGS from sFSGS, NC, and CKD of other etiologies with good accuracy. The generated pFSGS93 resulted in a sensitivity of 84.2% and specificity of 100% in the total cross-validated training data of pFSGS and sFSGS. Unfortunately, because of the low number of urine samples from pFSGS patients, these results could not be validated in an independent pFSGS cohort. However, the specificity of the pFSGS93 model could be validated in an independent cohort of 280 subjects and resulted in specificities of 99% for NC and 95% for other CKD etiologies.

As outlined above, current differentiation of pFSGS and sFSGS relies on the presence of nephrotic-range proteinuria and nephrotic syndrome. In our pFSGS cohort, the percentage of nephrotic-range proteinuria and nephrotic syndrome were 73.7% and 68.4%, respectively (Table 1). Classification of pFSGS was done regardless based on EM and clinical presentation. Two patients were missing proteinuria values at the time of biopsy but were exhibiting nephrotic-range proteinuria beforehand. Because of the missing data, nephrotic proteinuria could not be attributed at the time of biopsy. Another patient was just short of nephrotic-range proteinuria with a value of 3300 mg/d. The patient with nephrotic-range proteinuria who did not reach all criteria for nephrotic syndrome did not exhibit hyperlipidemia but met three out of four criteria for nephrotic syndrome. Taking this into consideration, the percentage of nephrotic-range proteinuria at time of biopsy might actually have been 89.5% (17/19).

Importantly, our cohort included sFSGS patients with various underlying diseases. The majority of patients were diagnosed with hypertensive nephropathy causative for the sFSGS, but the cohort included one patient each with bilateral renal hypoplasia, IgA nephropathy, collagen IV nephropathy, and pamidronate-induced collapsing FSGS. All these patients were identified as sFSGS. It is well-recognized that pamidronate-induced FSGS presents as the collapsing form of FSGS often associated with widespread FPE [13]. It is therefore reassuring that our classifier clearly recognized this case as secondary.

Our cohort also included one patient with morbid obesity and nephrotic-range proteinuria (20 g/d) who was suspected on clinical grounds to have sFSGS. Renal histology showed widespread FPE, and the patient was diagnosed as having pFSGS. Our classifier clearly grouped this patient into the pFSGS group with a very high score of +1.396.

Interestingly, one of the upregulated peptides in pFSGS included in pFSGS93 was Apo A-I. In the urine of pFSGS patients, abundance was up to 24-fold higher than in sFSGS patients and up to 21-fold higher than in patients with a mixture of different forms of CKD. No urinary excretion of Apo A-I was found in the urine of NC. As mentioned above, a high-molecular-weight form of Apo A-I (Apo A-Ib) was shown to be specifically present in the urine of recurrent-FSGS patients after kidney transplantation [50, 51]. It was even shown that urinary Apo A-Ib predated the recurrence in 4/5 episodes [51]. Clark *et al.* have shown elevated levels of a high-molecular-weight form of Apo A-I in urine of children with a relapse of MCGN or pFSGS but it was far more abundant in FSGS-relapsing patients. Patients in remission had levels similar to NC [52]. They also found Apo A-I to be elevated in proximal tubules of both MCGN- and FSGS-relapsing patients, but in FSGS, Apo A-I predominantly located in the brush border of the tubular cells and colocalized with the cubulin/megalin transporter [52]. Similarly, Jacobs-Cacha *et al.* could show that Apo A-I was predominantly localized at the brush border of tubular cells in patients with post-transplant recurrent FSGS, while in non-FSGS patients, it was found along the cytoplasm of the tubular cells [53]. The localization of Apo A-I at the brush border of tubular cells thus seems to be a specific feature of pFSGS in relapse and might also explain the increase in urinary excretion.

Other peptide fragments that were downregulated in pFSGS in comparison with sFSGS or NC originated from the PIGR, and the neurosecretory protein VGF. Peptides downregulated in pFSGS compared to sFSGS despite a significantly higher proteinuria might be of special interest in the understanding of pathophysiological aspect of the disease. Similar reductions of the PIGR were observed in severe COVID-19 but not in two other kidney diseases, diabetic nephropathy, and acute kidney injury [54].

Our study did not include patients with confirmed genetic causes of FSGS. Therefore, we cannot estimate how the pFSGS93 would classify genetic forms. Therefore, in selected cases genetic testing will still be necessary, as no biomarker test so far can identify genetic forms of FSGS. The vast majority of patients with monogenic forms of FSGS will not respond to corticosteroids and have a very low risk of recurrence in the allograft. Establishing a genetic cause thus avoids exposure to regimens used to treat pFSGS or to predict risk of post-transplantation recurrence. The likelihood of identifying a monogenic cause of FSGS correlates inversely with age. Mutations are identified in 60%–100% of children under the age of 1 year, whereas in adult-onset FSGS, a genetic cause was established in only 8%–14% [55–57]. Genetic testing should be performed for all patients who cannot be readily categorized by clinicopathological assessment and those resistant to steroid treatment. If genetic testing is done, a targeted next-generation sequencing approach with a large panel of genes known to be involved in FSGS is suggested.

Finally, patients with apparent sFSGS by biomarker testing and histology in the absence of defining disease characteristics leading to maladaptation may be unmasked as having collagen IV nephropathy, nephronophthisis [56, 58], or even Fabry's disease. In fact, in our study, one female FSGS patient had signs of a collagen type IV nephropathy with irregular thickness (143–849 nm) of an irregularly structured and laminated basement membrane. This patient was classified as having sFSGS by pFSGS93, underpinning the hypothesis of an underlying maladaptive process caused by mutations in collagen IV alpha 3, 4, or 5 genes.

Three of our patients with a pathology diagnosis of pFSGS were not recognized by pFSGS93 as such. One patient with a proteinuria of 10 g/d had a diagnosis of FSGS and IgA-nephropathy (MEST-C-Score: M1E0S1T0C0). The other two had proteinuria of 10.3 and 8.9 g/d and showed significant vascular changes suggestive of hypertensive nephropathy, and one of them additionally displayed signs of a mild immune complex GN by immunohistochemistry and electron microscopy. All three were classified as having pFSGS on the ground of widespread FPE. These three patients were not unequivocally patients with pFSGS. However, because our classification into pFSGS versus sFSGS was made by pathology on grounds of the extent of FPE, we decided to label them as pFSGS. Based on the biopsies that also showed IgA nephropathy and hypertensive nephropathy, the cases could very well have been classified as sFSGS indicating the potential benefit pFSGS93 could offer. Unfortunately, we do not have consistent information on treatment response or follow-up data to determine with more certainty if these patients in fact were cases of pFSGS or, as the pFSGS93 marker suggested, sFSGS.

### Limitations

Our pFSGS cohort did not receive testing for genetic forms of FSGS. Therefore, we cannot rule out for certain that the cohort included genetic FSGS cases as well. Furthermore, consistent follow-up data were not available to provide information on treatment responses and outcome of the patients. A further limitation was that the pFSGS could not be well matched to the sFSGS group regarding proteinuria, eGFR, and IFTA. This is, however, expected due to the different nature of these two forms of FSGS. However, the multiple regression analysis did not show association of eGFR and IFTA with the diagnosis of pFSGS. Moreover, additional analyses were performed to investigate whether the defined biomarkers are related to nephrotic-range proteinuria (data shown in pages 4–5 of Text S1 (see online supplementary material)). Additional patients with MCGN or membranous nephropathy (MN, *n* = 30, all with nephrotic-range proteinuria) that were matched for age, sex, IFTA, eGFR, and proteinuria to the pFSGS cohort were classified with the pFSGS93. This cohort was discriminated from the pFSGS cohort with an AUC of 0.83 (*P* < 0.0001). Furthermore, 45 of the 93 individual peptides included in the model also showed significant change regulation in the MCGN/MN cohort in accordance to the regulation observed in the sFSGS group. These observations indicate independence of the pFSGS93 biomarkers from eGFR or proteinuria. Because of the size of the cohort, validation of our biomarker on an independent pFSGS group was not possible. The biomarker panel requires further external validation.

## CONCLUSIONS

In conclusion, a urine peptide-based classifier that selectively discriminates pFSGS from sFSGS with 84% sensitivity and 100% specificity could be developed and is available for implementation. It could be of immediate value in instances where clinical presentation and histopathological findings are inconclusive in order to make therapeutic decisions. Furthermore, the biomarker could help guide diagnostic and therapeutic decisions in cases with contraindications to kidney biopsy. While specificity of 95%–99% could be confirmed in an independent sample, no additional samples from pFSGS patients were available for independent sensitivity validation. To support implementation, assessment of the classifier in an independent cohort of pFSGS patients would be beneficial.

Further studies of the urinary peptidome should focus not only on the differentiation between pFSGS and sFSGS but also on the prediction of a therapeutic response. In the face of the low number of FSGS cases, this will require a concerted action of several centers with well-described cases and follow-up data after therapeutic interventions.

## Supplementary Material

sfad296_Supplemental_Files

## Data Availability

Data will be made available upon request directed to the corresponding author. Proposals will be reviewed and approved by the investigators and collaborators based on scientific merit. After approval of a proposal, data will be shared through a secure online platform after signing the data access and confidentiality agreement.
