# Supplementary material for: Differentiating primary and secondary FSGS using non-invasive urine biomarkers
Source: Clin Kidney J. 2023 Dec 4;17(2):sfad296. doi: 10.1093/ckj/sfad296 (PMC10833144; doi:10.1093/ckj/sfad296)
Supplement: sfad296_Supplemental_Files [file sfad296_supplemental_files.zip › Supplementary Text_31082023.docx]

**Supplementary Text**

**Materials and Methods**

**Patient cohort**

Samples were collected on the day of the diagnostic kidney biopsy and before exposure to corticosteroids or other immunosuppressive therapies, with the following exceptions: one patient was permanently treated for rheumatoid arthritis with low dosage of corticosteroids, and in two patients the corticosteroid treatment was started before the sample collection (3 and 7 days, respectively). Clinical indication for biopsy was given beforehand independently of the study. Written consent for anonymized data retrieval and storage was obtained. The local ethics committee of the Friedrich-Alexander Universität Erlangen-Nürnberg provided approval for the nephrological biobank of the Klinikum Bayreuth (ethic approval code 264_20 B) and the urinary proteomics analysis (ethic approval code 221_20 B). Approval from the Ethics Committee of the Saxonian Board of Physicians, Dresden, Germany, was obtained for the study center Klinikum St. Georg Leipzig (ethic approval code EK-BR-14/20-1). On the day of the kidney biopsy, venous blood was drawn and immediately analyzed for creatinine concentration and estimated glomerular filtration rate (eGFR) using Chronic Kidney Disease Epidemiology Collaboration (EPI) equation [1]. Urine samples were obtained for assessment of proteinuria and cryo-stored by -80°C for further analysis. Biopsy cores from the study center in Bayreuth were sent to the Department of Nephropathology of the Friedrich-Alexander University Erlangen/Nürnberg and from the study center in Leipzig to the Nephropathology Department of the University Medical Center Hamburg-Eppendorf, Hamburg, for histopathological analysis.

A primary and, if applicable, a secondary histological diagnosis was extracted from the written histological report. Degree of interstitial fibrosis and tubular atrophy (IFTA) was determined visually after histopathological staining and given as percentage fibrotic vs. total interstitial area. The primary diagnosis based on both clinical and pathological criteria was used for definition of patient groups.

**Sample preparation and CE-MS analysis**

The prepared samples were analyzed using a P/ACE MDQ CE coupled on-line to a MicrOTOF II MS. A solution of 20% acetonitrile (Sigma-Aldrich, Taufkirchen, Germany) in HPLC-grade water (Merc, Darmstadt, Germany) supplemented with 0.94% formic acid (Merc KGaA, Darmstadt, Germany) was used as running buffer. The electrospray ionization (ESI) interface sprayer (Agilent Technologies, Palo Alto, CA, USA) was grounded, and the ion spray interface potential was set between −4 and −4.5 kV. Spectra were accumulated every 3 seconds over a range of mass-to-charge from 350 to 3000 m/z.

CE-MS data assessment was performed using the MosaiquesFinder software [2]. The mass spectral ion peaks representing identical molecules at different charge states were deconvoluted into single masses. Only signals with *z*>1 observed in a minimum of 3 consecutive spectra with a signal-to-noise ratio of at least 4 were considered. The resulting peak list characterizes each polypeptide by its mass and migration time. Data were calibrated utilizing 3151 internal standards as reference data points for mass and migration time by applying global and local linear regression, respectively. To correct for variability, a linear regression algorithm was applied for normalization, using internal standard peptides as reference [3]. Technical aspects have been described in more detail previously [4;5]. The obtained peak list characterizes each polypeptide by its calibrated molecular mass (Da), calibrated CE migration time (min), and normalized signal intensity.

**Sequencing of peptides**

MS/MS experiments were performed using an Ultimate 3000 nano-flow system (Dionex/LC Packings, Sunnyvale, CA, USA) or a P/ACE MDQ CE system (Beckman Coulter, Fullerton, CA), both connected to an LTQ Orbitrap hybrid MS (Thermo Fisher Scientific, Bremen, Germany) equipped with a nano-electrospray ion source. The MS is operated in data-dependent mode to automatically switch between MS and MS/MS acquisition. Survey full-scan MS spectra (from *m*/*z* 300–2000) were acquired in the Orbitrap. Ions were sequentially isolated for fragmentation. Data files were searched against the UniProt human nonredundant database using Proteome Discoverer 2.4 and the SEQUEST search engine without enzyme specificity (activation type: HCD; precursor mass tolerance: 5 ppm; fragment mass tolerance: 0.05 Da). No fixed modifications were selected, oxidation of methionine and proline were selected as variable modifications. For further validation of obtained peptide identifications, the correlation between peptide charge at the working pH of 2 and CE-migration time was utilized to minimize false-positive identification rates [6]. Here, the calculated CE-migration time of the sequence candidate, based on the number of basic amino acids with the sequence, was compared to the experimental migration time.

**Statistical analysis**

For the peptides used for the further analysis a peptide frequency threshold of at least 30% in one of the groups was considered. Wilcoxon rank-sum test was used for the calculation of the *P*-values. The *P*-values were adjusted for multiple testing assessed by the method described by Benjamini and Hochberg [7]. Potential biomarkers were combined in a support vector machine (SVM)-based classifier. SVM view a data point (probands urine sample) as a *p*-dimensional vector (*p* numbers of protein used), and they attempt to separate them with a (*p*-1) dimensional hyperplane. There are many hyperplanes that might classify the data. However, maximum separation (margin) between the two classes is of additional interest, and therefore, the hyperplane with the maximal distance from the hyperplane to the nearest data point is selected. All selected features are used without prior weighting to build up the *n*-dimensional classification space and to display the data set in the classification space. Classification itself (score estimation) is expressed as a numerical value quantifying the Euclidian distance of the data point to the maximal margin of the separation hyperplane among cases and controls in a multidimensional space.

To evaluate the accuracy of the generated model to discriminate the pFSGS and sFSGS groups receiver operating characteristic (ROC) curves were generated using MedCalc software (version 12.1.0.0; MedCalc Software, Mariakerke, Belgium). In an ROC, the sensitivity is plotted in function of 100-specificity for different cutoff points. Each point on the ROC represents a sensitivity/specificity pair corresponding to a particular decision threshold. The optimal balance of sensitivity and specificity was determined based on the Youden index J. The Youden index J [8] is defined as: *J* = max {sensitivity*c* + specificity*c* - 1} and gives the diagnostic threshold with the best sensitivity and specificity.

The diagnostic nomogram of pFSGS93 in combination with clinical variables was established using multiple linear regression analyses performed by MedCalc Software. Here the relationship between the diagnosis pFSGS vs. no pFSGS and pFSGS93, sex, age, proteinuria, eGFR, and IFTA was analyzed. All variables were entered into the model in one step. A variable was entered into the model if its associated significance level was *P*<.05.

**Results**

**Characteristics of patients**

The pFSGS group assessed at baseline included 13 (68.4%) males, had a mean age of 46.3±16.8 years, BMI of 31.0 kg/m^2^ [interquartile range (IQR) 27.0-33.3], systolic BP of 138±15 and diastolic BP of 85 mmHg (IQR 72.5-90). The sFSGS patient group included 30 (68.2%) males, had a significantly higher mean age of 57.6±16.7 years (*P*=.016), and showed no statistically significant differences in the following parameters: BMI, 28.7 kg/m^2^ (IQR 24.5-31.3 kg/m^2^); systolic BP, 140 mmHg (IQR 124-150 mmHg); and diastolic BP, 80 mmHg (IQR 70-90 mmHg). No significant differences between the pFSGS and sFSGS were also observed for the number of patients with diabetes and number of antihypertensive treatments. The pFSGS patients had a higher eGFR (median 56.0 mL/min/1.73 m²; IQR 37.4-94.9) and proteinuria (median 8.03 g/d; IQR 5.6-11.11); 14 of the pFSGS patients had nephrotic proteinuria] and lower IFTA (14.4±11.9 %) in comparison to sFSGS patients (medians for eGFR, proteinuria, and mean IFTA: 31.1 mL/min/1.73 m², 2.6 g/g Crea, and 28.3%, respectively).

Moreover, for the definition of pFSGS-specific peptides, further data from the human urinary database were extracted. Here, extracted were data of age- and sex-matched NC (n=98) with 73 (74.5%) males and a mean age of 44.7±15.4 years and of patients with various CKD (n=100) etiologies other than FSGS with 73 (73.0%) males and a mean age of 45.5±14.7 years. The characteristics of the patients used for definition of pFSGS-specific peptides are given in **Table 1**. The list of the CKD etiologies with the number of patients is given in **Table 2**.

**Analysis of covariables and nomogram generation**

The multiple regression analysis that was used to estimate which parameter is associated with the diagnosis of pFSGS resulted in two significant associated parameters: pFSGS93 and proteinuria. The results of multiple regression are shown in **Table S2**.

**Table S2**. The regression equation table. For each variable the different regression coefficients with standard error, *r*_partial_, *t*-value and *P*-value are given.

| **Independent variables** | **Coefficient** | **Std. Error** | ***r*_partial_** | ***t*** | ***P*** |
| --- | --- | --- | --- | --- | --- |
| **(Constant)** | 0.35750000 |  |  |  |  |
| **IFTA (%)** | 0.00026090 | 0.001247 | 0.01768 | 0.209 | 0.8346 |
| **Age** | -0.00054080 | 0.001470 | -0.03109 | -0.368 | 0.7134 |
| **eGFR (CKD-EPI), ml/min/1.73m²** | 0.00082580 | 0.0008013 | 0.08677 | 1.031 | 0.3045 |
| **Proteinuria, g/d** | -0.00002488 | 0.000005705 | -0.3459 | -4.361 | <0.0001 |
| **pFSGS93** | 0.20310000 | 0.02383 | 0.5844 | 8.522 | <0.0001 |
| **Sex** | 0.038820000 | 0.04592 | 0.07126 | 0.845 | 0.3994 |

**Comparison of pFSGS and MCD/MN Using pFSGS93**

We extracted from the database a further 30 urinary peptide datasets of age, sex, IFTA, eGFR, and proteinuria matched to pFSGS cohort minimal change disease (MCD, n=11) and membranous nephritis (MN, n=19) patients with nephrotic syndrome and classified these patients using pFSGS93. ROC analysis of the pFSGS and MCD/MN cohorts resulted in an AUC of 0.83 (*P*<0.0001, **Figure S2**). In addition, the signal intensities of the 93 defined biomarkers in the MCD/MN cohort were investigated and compared to the signal intensities observed in the pFSGS group. Of the 93 peptide biomarkers, 45 showed significant change in the MCD/MN group vs. pFSGS group. The regulation of these peptides in the MCD/MN group was analogue to sFSGS, other CKD etiologies and NC groups.


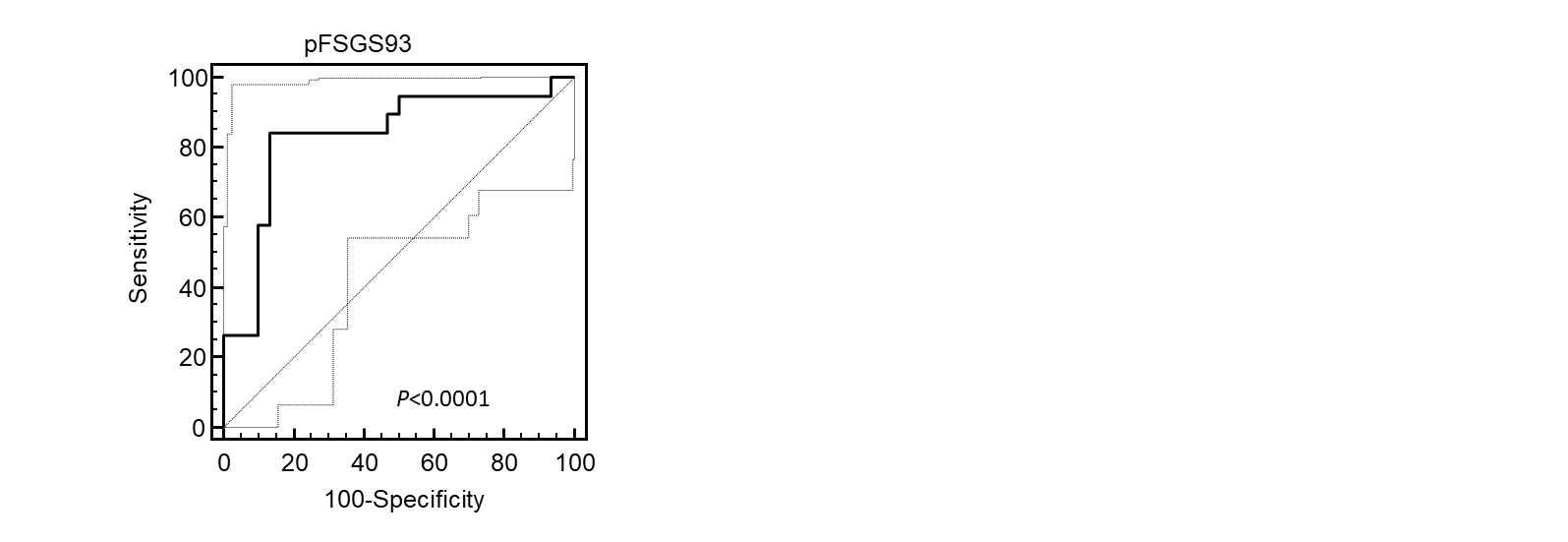


**Figure S1**. ROC analysis of MCD (n=11) and MN (n=19) vs. pFSGS (n=19) using pFSGS93 classifier.

**Discussion**

Gene expression analysis of common podocyte proteins (ACTN4, GLEPP-1, WT-1, synaptopodin, dystroglycan, nephrin, podoplanin, and podocin) in microdissected glomeruli suggested that the podocin:synaptopodin expression ratio could be useful to distinguish FSGS from minimal change disease (MCD). However, no differentiation could be achieved from membranous GN, and it is not reported whether primary or secondary FSGS forms were investigated [9]. Kidney microRNA expression has been analyzed in FSGS. Several miRNAs were upregulated in glomeruli of pFSGS patients [10], and miRNA-193a might be relevant for development of the disease, as it decreases the expression of WT-1, which compromises podocyte function [11]. Interestingly, miRNA-193a is also increased in urinary exosomes of children with pFSGS, compared to MCD patients [12], which might make this marker amenable for urinary diagnostics. The validity of this marker to differentiate between primary and secondary FSGS was, however, not investigated. The search for plasma biomarkers has focused on the putative permeabilizing factor. One of the most promising candidates was suPAR, which was found to be increased in serum of pFSGS patients [13]. Injection of recombinant uPAR into a knockout mouse model induced proteinuria [13]. Unfortunately, later studies were unable to reproduce these results in independent cohorts [14-16]. It was also shown that suPAR levels increased with decreasing eGFR. In fact suPAR levels above the suggested cutoff for FSGS were found in 88% and 95% of non-FSGS patients with an eGFR between 30 and 45 mL/min/1.73 m^2^ or <30 mL/min/1.73 m^2^, respectively [17]. Urinary levels of suPAR have also been studied and were shown to be elevated in primary FSGS, but they were also increased, though to a lesser degree, in MCD, membranous nephropathy or secondary FSGS [18]. This makes it unlikely that suPAR is the true permeabilizing factor in primary FSGS.

It has been shown that plasma of relapsing FSGS patients induces the expression of specific genes when added to cultured podocytes. As an example, interleukin-1beta gene expression induced by serum of recurrent FSGS patients shows >80% sensitivity and specificity to discriminate relapsing FSGS from other nephropathies [19]. Such an assay, however, is labor and time intensive, since it requires cultivation of podocytes.

Urine has been used in the past with the aim to distinguish different forms of glomerulopathies. Varghese et al. separated urine proteins by 2-dimensional electrophoresis in 32 patients with FSGS, lupus nephritis, membranous nephropathy and diabetic nephropathy and protein abundances were used to create a prediction algorithm. The model predicted the presence of the respective diseases with sensitivities between 75% and 86% and specificities from 67% to 92% [20]. No differentiation between primary and secondary FSGS was made. There is only one biomarker that has been specifically associated with FSGS, in particular with post-transplantation recurrent FSGS. This is apolipoprotein A-Ib (Apo A-Ib), a modified form of Apo-I. In a total of 119 patients studied, the Apo-Ib form was detected in 13 of 14 relapsing patients, but in only 1 of the 61 nonrelapsing patients and in 1 of 30 patients with FSGS-unrelated proteinuria [21]. Kalantari et al. tried to establish a urinary biomarker for steroid-resistant and steroid-sensitive FSGS using high-resolution mass spectrometry and identified 21 proteins as discriminating species. The most drastic fold changes were observed for Apo A-I and Matrix-remodeling protein 8 [22]. The same group tried to find a diagnostic biomarker for FSGS by comparing FSGS patients with NC and patients with IgA nephropathy. A total of 77 proteins were considered as putative biomarkers of FSGS. The most significant differentially expressed proteins were CD59, CD44, IBP7, Robo4, and DPEP1, proteins involved in complement pathway, sclerosis, cell proliferation, actin cytoskeleton remodeling, and activity of TRPC6 [23]. But again, no attempt was made to differentiate primary from secondary FSGS.

The only biomarker study that tested the discriminatory power between primary and secondary FSGS was a study that measured urinary CD80 excretion in various glomerular diseases in 411 patients from the Mayo Clinic and the Nephrotic Syndrome Study Network Consortium (NEPTUNE). CD80 plays a role in innate and adaptive immunity activation and overexpression of CD80 in podocytes led to slit diaphragm protein rearrangement in vitro and proteinuria in animal models [24;25]. Urinary CD80 excretion was significantly lower in secondary than in pFSGS. However, sensitivity (75%) and specificity (75%) were quite low at a cutoff of CD80/creatinine of 42.1 ng/g [26].

Despite all these studies, none of these features is pathognomonic for primary or secondary FSGS and identifying pFSGS continues to be challenging. After all, FSGS is a histological pattern rather than a specific disease. This is further aggravated by the low prevalence of the disease and the lack of clinical tools for its risk stratification, prediction of remission, treatment selection, and drug response.

Therefore, there is an urgent need for a nongenetic biomarker that discriminates among FSGS types and allows classification of patients.

**References**

Reference List

1. Levey AS, Stevens LA, Schmid CH *et al*. A new equation to estimate glomerular filtration rate. *Ann Intern Med* 2009; 150: 604-612

2. Latosinska A, Siwy J, Mischak H, Frantzi M. Peptidomics and proteomics based on CE-MS as a robust tool in clinical application: The past, the present, and the future. *Electrophoresis* 2019; 40: 2294-2308

3. Jantos-Siwy J, Schiffer E, Brand K *et al*. Quantitative Urinary Proteome Analysis for Biomarker Evaluation in Chronic Kidney Disease. *J Proteome Res* 2009; 8: 268-281

4. Mavrogeorgis E, Mischak H, Latosinska A, Siwy J, Jankowski V, Jankowski J. Reproducibility Evaluation of Urinary Peptide Detection Using CE-MS. *Molecules* 2021; 26:

5. Mischak H, Vlahou A, Ioannidis JP. Technical aspects and inter-laboratory variability in native peptide profiling: The CE-MS experience. *Clin Biochem* 2013; 46: 432-443

6. Zürbig P, Renfrow M.B., Schiffer E *et al*. Biomarker discovery by CE-MS enables sequence analysis via MS/MS with platform-independent separation. *Electrophoresis* 2006; 27: 2111-2125

7. Dakna M, Harris K, Kalousis A *et al*. Addressing the challenge of defining valid proteomic biomarkers and classifiers. *BMC Bioinformatics* 2010; 11: 594

8. YOUDEN WJ. Index for rating diagnostic tests. *Cancer* 1950; 3: 32-35

9. Schmid H, Henger A, Cohen CD *et al*. Gene expression profiles of podocyte-associated molecules as diagnostic markers in acquired proteinuric diseases. *J Am Soc Nephrol* 2003; 14: 2958-2966

10. Baker MA, Davis SJ, Liu P *et al*. Tissue-Specific MicroRNA Expression Patterns in Four Types of Kidney Disease. *J Am Soc Nephrol* 2017; 28: 2985-2992

11. Gebeshuber CA, Kornauth C, Dong L *et al*. Focal segmental glomerulosclerosis is induced by microRNA-193a and its downregulation of WT1. *Nat Med* 2013; 19: 481-487

12. Huang Z, Zhang Y, Zhou J, Zhang Y. Urinary Exosomal miR-193a Can Be a Potential Biomarker for the Diagnosis of Primary Focal Segmental Glomerulosclerosis in Children. *Biomed Res Int* 2017; 2017: 7298160

13. Wei C, El HS, Li J *et al*. Circulating urokinase receptor as a cause of focal segmental glomerulosclerosis. *Nat Med* 2011; 17: 952-960

14. Maas RJH, Wetzels JFM, Deegens JKJ. Serum-soluble urokinase receptor concentration in primary FSGS. *Kidney Int* 2012; 81: 1043-1044

15. Spinale JM, Mariani LH, Kapoor S *et al*. A reassessment of soluble urokinase-type plasminogen activator receptor in glomerular disease. *Kidney Int* 2015; 87: 564-574

16. Wada T, Nangaku M, Maruyama S *et al*. A multicenter cross-sectional study of circulating soluble urokinase receptor in Japanese patients with glomerular disease. *Kidney Int* 2014; 85: 641-648

17. Hayek SS, Sever S, Ko YA *et al*. Soluble Urokinase Receptor and Chronic Kidney Disease. *N Engl J Med* 2015; 373: 1916-1925

18. Huang J, Liu G, Zhang YM *et al*. Urinary soluble urokinase receptor levels are elevated and pathogenic in patients with primary focal segmental glomerulosclerosis. *BMC Med* 2014; 12: 81

19. Srivastava P, Solanki AK, Arif E *et al*. Development of a novel cell-based assay to diagnose recurrent focal segmental glomerulosclerosis patients. *Kidney Int* 2019; 95: 708-716

20. Varghese SA, Powell TB, Budisavljevic MN *et al*. Urine biomarkers predict the cause of glomerular disease. *J Am Soc Nephrol* 2007; 18: 913-922

21. Lopez-Hellin J, Cantarell C, Jimeno L *et al*. A form of apolipoprotein a-I is found specifically in relapses of focal segmental glomerulosclerosis following transplantation. *Am J Transplant* 2013; 13: 493-500

22. Kalantari S, Nafar M, Rutishauser D *et al*. Predictive urinary biomarkers for steroid-resistant and steroid-sensitive focal segmental glomerulosclerosis using high resolution mass spectrometry and multivariate statistical analysis. *BMC Nephrol* 2014; 15: 141

23. Nafar M, Kalantari S, Samavat S, Rezaei-Tavirani M, Rutishuser D, Zubarev RA. The novel diagnostic biomarkers for focal segmental glomerulosclerosis. *Int J Nephrol* 2014; 2014: 574261

24. Khullar B, Balyan R, Oswal N *et al*. Interaction of CD80 with Neph1: a potential mechanism of podocyte injury. *Clin Exp Nephrol* 2018; 22: 508-516

25. Reiser J, von GG, Loos M *et al*. Induction of B7-1 in podocytes is associated with nephrotic syndrome. *J Clin Invest* 2004; 113: 1390-1397

26. Gonzalez Guerrico AM, Lieske J, Klee G *et al*. Urinary CD80 Discriminates Among Glomerular Disease Types and Reflects Disease Activity. *Kidney Int Rep* 2020; 5: 2021-2031
